# Novel disease-causing variant in *RDH12* presenting with autosomal dominant retinitis pigmentosa

**DOI:** 10.1136/bjophthalmol-2020-318034

**Published:** 2021-05-24

**Authors:** Manickam Nick Muthiah, Angelos Kalitzeos, Kate Oprych, Navjit Singh, Michalis Georgiou, Genevieve Ann Wright, Anthony G Robson, Gavin Arno, Kamron Khan, Michel Michaelides

**Affiliations:** 1 Cell and Gene Therapy, University College London Institute of Ophthalmology, London, UK; 2 Vitreoretinal Research, Moorfields Eye Hospital NHS Foundation Trust, London, UK; 3 Moorfields Eye Hospital NHS Foundation Trust, London, UK; 4 Great Ormond Street Hospital For Children NHS Trust, London, UK; 5 National Institute for Health Research Biomedical Research Centre at Moorfields Eye Hospital NHS Foundation Trust and UCL Institute of Ophthalmology, London, Greater London, UK; 6 Electrophysiology, Moorfields Eye Hospital NHS Foundation Trust, London, UK; 7 Department of Ophthalmology, Leeds Teaching Hospitals NHS Trust, Leeds, UK; 8 Department of Ophthalmology, Calderdale and Huddersfield NHS Foundation Trust, Huddersfield, West Yorkshire, UK

**Keywords:** retina, dystrophy, genetics, imaging

## Abstract

**Aim:**

To describe the clinical and molecular features of a novel, autosomal dominant *RDH12*-retinopathy.

**Methods:**

Retrospective cross-sectional study. Twelve individuals from a four-generation British pedigree underwent ophthalmic examination, genotyping using next generation sequencing, including whole genome sequencing and multimodal retinal imaging including fundus photography, optical coherence tomography (OCT), autofluorescence imaging and adaptive optics (AO) scanning light ophthalmoscopy were performed. Visual electrophysiology was performed in a subset.

**Results:**

Eight family members were confirmed as affected by genotyping heterozygous for *RDH12* c.763delG. Visual acuity ranged from −0.1 to 0.2 logMAR. Affected individuals had constricted visual fields. A parafoveal and peripapillary ring of hyper-autofluorescence was seen initially, and with progression the area of perifoveal hypo-autofluorescence increased to involve the parafoveal area. Mild retinal thinning was identified on OCT imaging with reduction in both foveal total retinal and outer nuclear layer thickness. Cone densities along the temporal meridian were reduced in affected individuals compared with normative values at all temporal eccentricities studied. One individual with incomplete penetrance, was identified as clinically affected primarily on the basis of AO imaging. Full-field electroretinography demonstrated a rod-cone pattern of dysfunction and large-field pattern electroretinography identified peripheral macular dysfunction.

**Conclusions:**

This novel heterozygous variant *RDH12* c.763delG is associated with a rod-cone dystrophy with variable expression. Determination of the degree of penetrance may depend on the modality employed to phenotypically characterise an individual. This rare and specific heterozygous (dominant) variant is predicted to result in a gain of function, that causes disease in a gene typically associated with biallelic (recessive) variants.

## Introduction

The human visual cycle depends on the metabolism and transport of vitamin A molecules (retinoids) in both photoreceptors and the retinal pigment epithelium (RPE). Photons of light, captured in the photoreceptor outer segment, isomerise 11-*cis*-retinal to all-*trans*-retinal, which then requires recycling to restore 11-*cis*-retinal. RPE65, the most well-known enzyme in this pathway, catalyses one critical step in regenerating 11-*cis*-retinal in the RPE,^1–3^ and loss of enzymatic function is associated with an early-onset rod-cone dystrophy (Leber congenital amaurosis, LCA type 2).^4–6^ *RPE65* gene replacement therapy is now available to treat this.^7^ Another enzyme vitally important to retinoid recycling, present in the photoreceptors, is retinol dehydrogenase 12 (RDH12, LCA type 13).^8 9^ It functions as a retinal reductase, with highest affinity for all-*trans*-retinal, metabolising it in the photoreceptor to generate all-*trans*-retinol prior to transport to the RPE.^10 11^ It may also have an additional role in the detoxification of lipid peroxidation products.^10 12^ As with RPE65, loss of RDH12 function is associated with an early-onset retinal dystrophy.^13–15^ Importantly, heterozygous carriers of loss of function alleles are entirely asymptomatic, suggesting that a single functional allele is sufficient.

Occasionally, rather than causing retinal disease by biallelic loss of function, specific variants may confer a dominant, gain of function. This phenomenon has been observed for a number of LCA genes including *GUCY2D* and a rare *RPE65* allele (p.Asp477Gly), which has been observed segregating with a milder, later-onset retinal dystrophy, and transmitted in an autosomal dominant manner.^16 17^ In addition, a single large pedigree has been reported segregating a rare, heterozygous *RDH12* allele (c.778delG, p.Glu260Argfs*18 formerly annotated as c.776delG).^18^ More recently, two further unrelated families were identified who harbour the rare heterozygous *RDH12* variant c.759delC;(p.Phe254Leufs*24) and a retinal dystrophy phenotype.^19^ The current work presents a detailed characterisation of the clinical and molecular features observed in a fourth, four-generation British family harbouring a novel, dominant form of *RDH12*-retinopathy (c.763delG; (p.Val255Serfs*23)).

## Material and methods

### Patient identification

Patients with a diagnosis of rod-cone dystrophy and a single, heterozygous, plausibly pathogenic variant in *RDH12* were identified from a single family attending Moorfields Eye Hospital, UK. Informed consent was obtained from all patients and unaffected family members (12 individuals in total).

All individuals were examined by slit-lamp biomicroscopy. Retinal structure and function were assessed in more detail in a subset (see below). Individuals were defined as affected if they were symptomatic (reported any degree of nyctalopia with or without constriction of peripheral visual fields) and had any evidence of a rod-cone dystrophy (either intraretinal pigment migration, retinal arteriolar attenuation, outer retinal atrophy or autofluorescence changes).

### Genomic studies

**Figure 1:**
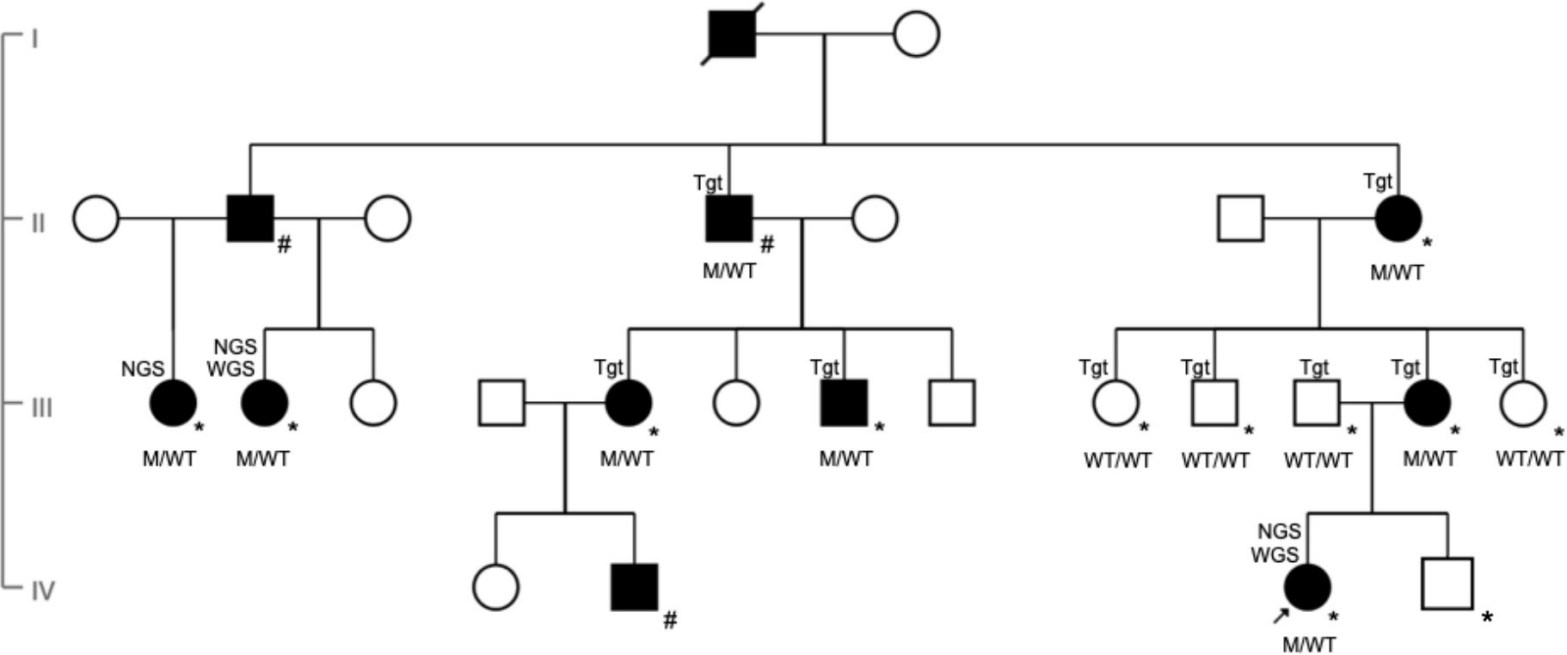
Pedigree (GC21021) showing affected index patient (black arrow) and family members over four generations (denoted as M/WT for heterozygotes and WT/WT for those with wild type alleles). Individuals clinically affected with autosomal dominant retinitis pigmentosa are represented in black symbols while unaffected individuals with open symbols. Deceased individual is represented with a slash. Squares represent men and circles represent women. M, c.763delG (p.Val255Serfs*23); WT, wild type. All those examined at Moorfields are marked with an *. Individuals examined at other institutions are marked with a #. Individuals identified as affected by whole genome sequencing, next generation sequencing and targeted sequencing are labelled WGS, NGS and Tgt, respectively, in the pedigree.

The index case (figure 1, IV-3) underwent panel-based next generation sequencing, targeting 105 genes known to cause retinal disease. Later testing in members of the extended family (n=2), (figure 1, III-1, III-2) used an updated version of the same retinal dystrophy panel, which this time screened 176 genes. All testing was performed in a National Genetics Reference Laboratory with expertise in rare disease (Manchester University NHS Foundation Trust, UK). Concurrently, one individual (IV-3) underwent whole genome sequencing (WGS) as part of the National Institute for Health Research BioResource – Rare Diseases study^20^ and previous individual (III-2) underwent WGS as part of the Genomics England 100 000 genomes pilot study.^21^ All other family members (n=9, including the index patient’s parents, III-11 and III-12) underwent bidirectional Sanger sequencing for the identified variant in *RDH12*.

### Retinal imaging

Colour fundus photography was performed with TRC-50IX (Topcon Medical Systems, Paramus, New Jersey). Spectral domain optical coherence tomography (SD-OCT) horizontal, volume (25 B-scans) and/or line scans covering either 20° or 30° were obtained in six family members (Heidelberg Engineering, Heidelberg, Germany). These were automatically registered to simultaneously acquired near-infrared (815 nm) reflectance scanning laser ophthalmoscope fundus images. Automated real-time tracking with at least nine scans was used. The foveal total retinal thickness (TRT) and outer nuclear layer thickness (ONLT) in both eyes were measured in five family members by means of the vendor supplied Heidelberg Eye Explorer (HEYEX) software (V.1.9.10.0) by a single, experienced grader (AK). The TRT was defined as the distance between the internal limiting membrane and the RPE/Bruch’s membrane, while the ONLT was measured as the distance between the outer plexiform layer and the external limiting membrane, following semi-automated segmentation. Longitudinal analysis of foveal TRT was performed in two affected family members in order to ascertain progression. Fundus short wavelength autofluorescence (486 nm) imaging (Heidelberg Engineering, Heidelberg, Germany) was performed in a subset of the family members and follow-up images were spatially registered to the baseline ones to aid comparison.

### Adaptive optics scanning light ophthalmoscopy

Photoreceptor mosaic imaging using a custom-built adaptive optics scanning light ophthalmoscope (AOSLO) with both confocal and non-confocal split-detection capabilities,^22 23^ was performed on the affected index patient, her minimally symptomatic mother and her unaffected father. A temporal strip was recorded on all three family members, from the foveal centre through to at least 5° temporally. Image sequences were processed and montaged using custom software.^24 25^ For each eccentricity studied, a minimum of two, and a maximum of three 55 µm^2^ square regions of interest (ROIs), were cropped and photoreceptors were marked by a single, experienced grader (NS).^26 27^ Cone densities were calculated by dividing the number of bound cells within each ROI over the area encompassed by them.^27^

### Visual electrophysiology

Full-field electroretinography and pattern electroretinography (PERG) were performed on the index patient and the first cousin once removed (IV-3 and III-2, respectively) using protocols that incorporated the International Society of Clinical Electrophysiology of Vision standards,^28 29^ using gold foil corneal recording electrodes. The PERGs were recorded to both a standard chequerboard field size (15×11 degrees) and additionally to a large field stimulus (30×22 degrees) according to a previously described method.^30^

## Results

### Genetic analysis

Gene panel-based testing on three individuals (III-1, III-2 and IV-3) identified a single likely pathogenic variant as the likely cause of autosomal dominant retinitis pigmentosa (adRP) in those individuals (GRCh38 chr14:67 729 295del: NM_152443.3: c.763delG, (p.Val255Serfs*23)). Subsequent WGS analysis (IV-3) confirmed the absence of additional coding, non-coding or structural variants in known inherited retinal disease genes or likely pathogenic variants in genes previously not associated with retinal diseases. Family testing identified a total of eight individuals (labelled as M/WT in figure 1, excluding the deceased) from this extended family who were heterozygous for *RDH12* c.763delG.

### Clinical phenotype

In total, 12 individuals were examined. Clinical characteristics of the seven individuals harbouring the rare variant in *RDH12* are presented in table 1. Six manifested signs of a rod-cone dystrophy, with an age range of 12–72 years (at time of this study). These six first noted nyctalopia in their early-to-mid teenage years. Visual acuity ranged from −0.1 to 0.2 logMAR (median 0.00 logMAR). Reduced acuity (0.2 logMAR) appeared to be associated with macular oedema, rather than duration of disease (patient III-1). Goldmann kinetic perimetry identified constriction of peripheral visual fields, which, in the most severely affected individual was reduced to the central 5°–10° (patient III-1), with the index patient (IV-3) having significant constriction, especially of the temporal field.

**Table 1:** Phenotype findings in affected family members

Family members	Age (y)	VA OD (logMAR)	VA OS (logMAR)	VF	Fundus OD and OS	OCT	AF	Full-field ERG
IV-3	12	0	0	Const	Bone spicules Peripheral RPE atrophy	Peripheral photoreceptor degeneration, asymmetric remnant island around fovea extending nasally	Parafoveal asymmetric ring of hyper-AF	mild reduction in DA and LA, LF-PERG
IV-2	18	0.10	0	na	Bone spicules	na	na	mild reduction in DA and LA
III-12	40	0	0	Normal	Unremarkable	Macular ONL thinning	Localised region of hyper-AF temporal macula in OS only	Normal
III-5	43	0.1	0.1	Nasal const +mid peripheral ring scotoma	Bone spicules	na	na	mild reduction in DA and LA
III-2	39	−0.1	−0.1	Const	Bone spicules Peripheral RPE atrophy	Macular ONL thinning	Parafoveal ring of hyper-AF	OD slight reduction in DA and LA compared with OS normal. OU slight reduction LF-PERG
III-1	29	0.20	0.20	Const <10	Bone spicules Peripheral RPE atrophy	Perifoveal/parafoveal photoreceptor degeneration and CMO	Parafoveal ring of hyper-AF	na
II-7	72	0.1	0.1	Multiple central scotomata	Bone spicules Peripheral RPE atrophy	Parafoveal photoreceptor degeneration	Extensive parafoveal and temporal macula hypo-AF	na

AF, autofluorescence; CMO, cystoid macular oedema; Const, Constricted; DA, dark-adapted; ERG, electroretinogram; LA, light-adapted; LF-PERG, large field pattern ERG; na, not available; OCT, optical coherence tomography; OD, oculus dextra; ONL, outer nuclear layer; OS, oculus sinistra; OU, oculus uterque; RPE, retinal pigment epithelium; VA, visual acuity; VF, visual field; y, years.

**Figure 2:**
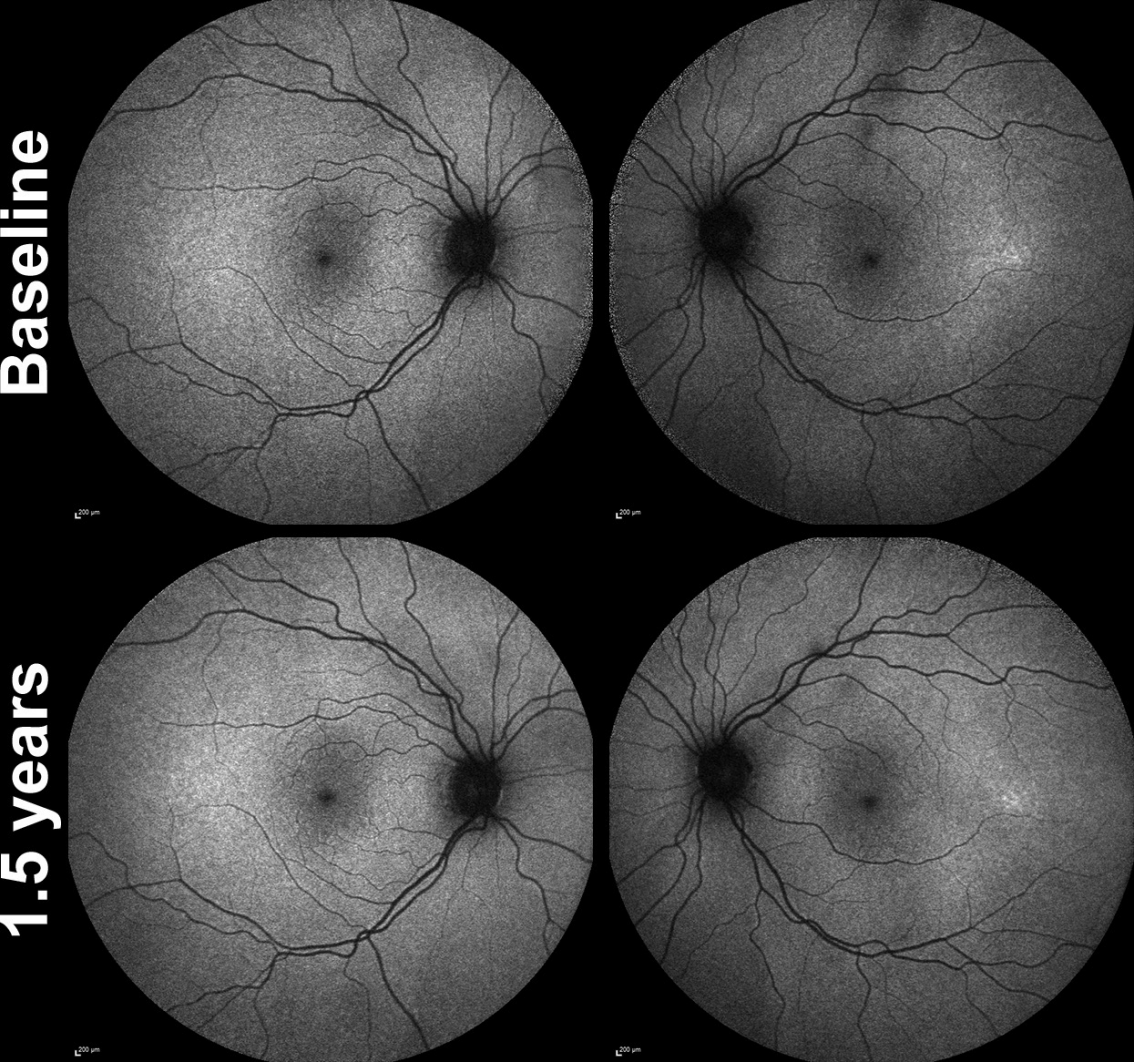
Longitudinal fundus autofluorescence (FAF) imaging in minimally symptomatic mother of the index patient (III-12) over a 1.5 years follow-up. Age at baseline was 39 years and 3 months old. Focal area of hyper-autofluorescence temporal macula of left eye and no change noted in this subtle hyper-autofluorescence over the 1.5 years of follow-up. The FAF imaging of right eye was unremarkable.

One individual (III-12) was minimally symptomatic (uniocularly) at the age of 40, with no evidence of pigment migration into the retina. Rather unusually, she had self-identified an asymmetry in scotopic visual function, commenting that in dim light, she could see better out of her right eye compared with her left. Macular structure as assessed by SD-OCT qualitatively appeared normal, however AOSLO imaging revealed a reduction in cone densities (online supplemental file 7) at all ROIs temporal to the fovea compared with normative data,^27^ while autofluorescence imaging showed a subtle hyper-autofluorescent spot in the left eye around 14° temporal to the fovea which did not change across 1.5 years of follow-up (figure 2). Visual electrophysiology was normal.

10.1136/bjophthalmol-2020-318034.supp7 Supplementary data

**Figure 3:**
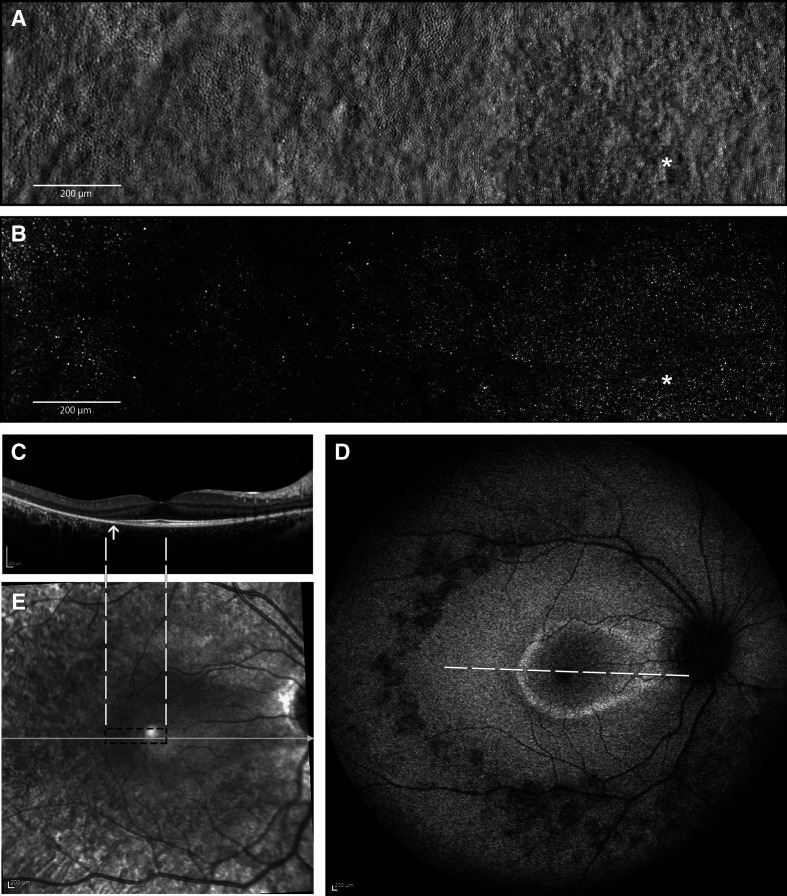
Multimodal imaging of the right eye of the affected index patient (IV-3) at the age of 14 years. (A) Split-detection AOSLO from the fovea (white asterisk) out to 5° temporally capturing the en face cone photoreceptor inner segments; (B) confocal AOSLO from the fovea (white asterisk) out to 5° temporally capturing the en face cone photoreceptor outer segments; (C) horizontal transfoveal OCT line scan shows loss of inner segment ellipsoid band and outer retinal loss (indicated by arrow). Dashed white lines indicate the location and extent of the AOSLO en face images; (D) fundus autofluorescence image centred on the fovea 55° wide. A ring of parafoveal hyper-autofluorescence, patchy perifoveal hypo-autofluorescence, scattered areas of hypo-autofluorescence along the temporal arcades and in mid-peripheral retina. White dashed line indicates the location and extent of the OCT scan in C; (E) corresponding near infrared reflectance image of the OCT scan (white line) in C. Black dashed rectangle indicates the location and extent of the AOSLO en face images in A and B. AOSLO, adaptive optics scanning light ophthalmoscope; OCT, optical coherence tomography.

Five additional members of the family were examined. All were asymptomatic, and had a normal eye examination. The rare *RDH12* variant was not identified in four of these individuals who underwent genotyping.

Three other individuals were confirmed from clinical records to have been diagnosed with rod-cone dystrophy, but were not available for examination. One of these three individuals consented for their sample to be genetically tested and the rare *RDH12* variant was also identified.

### Retinal imaging

The clinical phenotype of the affected index patient and her minimally symptomatic mother are shown in figures 2 and 3, respectively. Index patient’s (IV-3) fundus autofluorescence (FAF) image of right eye (figure 3D) centred on the fovea shows areas of parafoveal ring of hyper-autofluorescence along with an area of perifoveal hypo-autofluorescence and an area of extensive hypo-autofluorescence along the supero-temporal and infero-temporal temporal arcades up to the temporal edge of the macula which in fact correlates to the area of mild pigment migration into the retina (mild bone spicule) on colour fundus (which is not included here). The parafoveal ring of hyper-autofluorescence correlates to the area of inner segment ellipsoid band and outer retinal loss as noted on the transfoveal OCT line scan (figure 3C, D). Split-detection (figure 3A) and confocal AOSLO imaging (figure 3B) from the fovea (white asterisk) out to 5° temporally captured the en face cone photoreceptor inner and outer segments, respectively. The mother had a subtle change noted on FAF imaging of her left eye (figure 2) with a focal area of hyper-autofluorescence at the temporal macula and over the 1.5 years of follow-up there was no change in this focal hyper-autofluorescence. FAF imaging of the right eye was unremarkable.

### Quantitative OCT analysis

The foveal TRT and ONLT in both eyes for age-matched OCT of children siblings and age-similar adult family members are shown in (online supplemental file 1). In the younger affected family member, index patient IV-3 has a 5.3%–6.1% and 3.4%–4.0% reduction in foveal TRT and ONLT, respectively, compared with age-matched OCT of unaffected brother IV-4. The older affected family members III-2 and III-12 have a 5.7%–16.6% and 15.1%–20% reduction in foveal TRT and ONLT, respectively, compared with age-similar unaffected individual III-11.

10.1136/bjophthalmol-2020-318034.supp1 Supplementary data

Longitudinal data on the index patient (IV-3) from the age of 10 years over 4.75 years, and extended family member III-2 from the age of 36 over a 7.5-year period are shown in online supplemental files 2 and 3, respectively. The former shows a gradual increase in foveal TRT in right eye (12 µm) and left eye (6 µm); and the latter a decrease in foveal TRT in right (6 µm) and left eye (4 µm); likely to indicate relative stability of structure.

10.1136/bjophthalmol-2020-318034.supp2 Supplementary data

10.1136/bjophthalmol-2020-318034.supp3 Supplementary data

### FAF

Short-wavelength FAF was abnormal in all affected family members examined who harboured the *RDH12* variant, and unremarkable in all unaffected individuals with imaging available. Qualitative changes included a ring of parafoveal hyper-autofluorescence, patchy perifoveal hypo-autofluorescence, scattered areas of hypo-autofluorescence in mid-peripheral retina and a peripapillary ring of hyper-autofluorescence. All affected individuals had peripapillary sparing.

The mildest change was present in the index patient’s minimally symptomatic mother (III-12), as a subtle region of hyper-autofluorescence in the peripheral macula, evident in the temporal macula of the symptomatic left eye only (table 1 and figure 2). This did not appear to progress during the 1.5-year follow-up period.

The most severe changes were present in the oldest patient, the index patient’s affected maternal grandmother II-7, whose FAF showed extensive parafoveal and temporal macula hypo-autofluorescence bilaterally in area where the outer retina had been lost as noted on the corresponding OCT scans (online supplemental file 4). Analysis of longitudinal imaging data from the index patient (IV-3) over 3 years (online supplemental file 5), and extended family member III-2 over a 7.5-year period (online supplemental file 6), highlights a gradual constriction of the parafoveal hyper-autofluorescent rings and an increase in the area of perifoveal hypo-autofluorescence at the temporal macula in both eyes.

10.1136/bjophthalmol-2020-318034.supp4 Supplementary data

10.1136/bjophthalmol-2020-318034.supp5 Supplementary data

10.1136/bjophthalmol-2020-318034.supp6 Supplementary data

### AOSLO

#### Cone density

**Figure 4:**
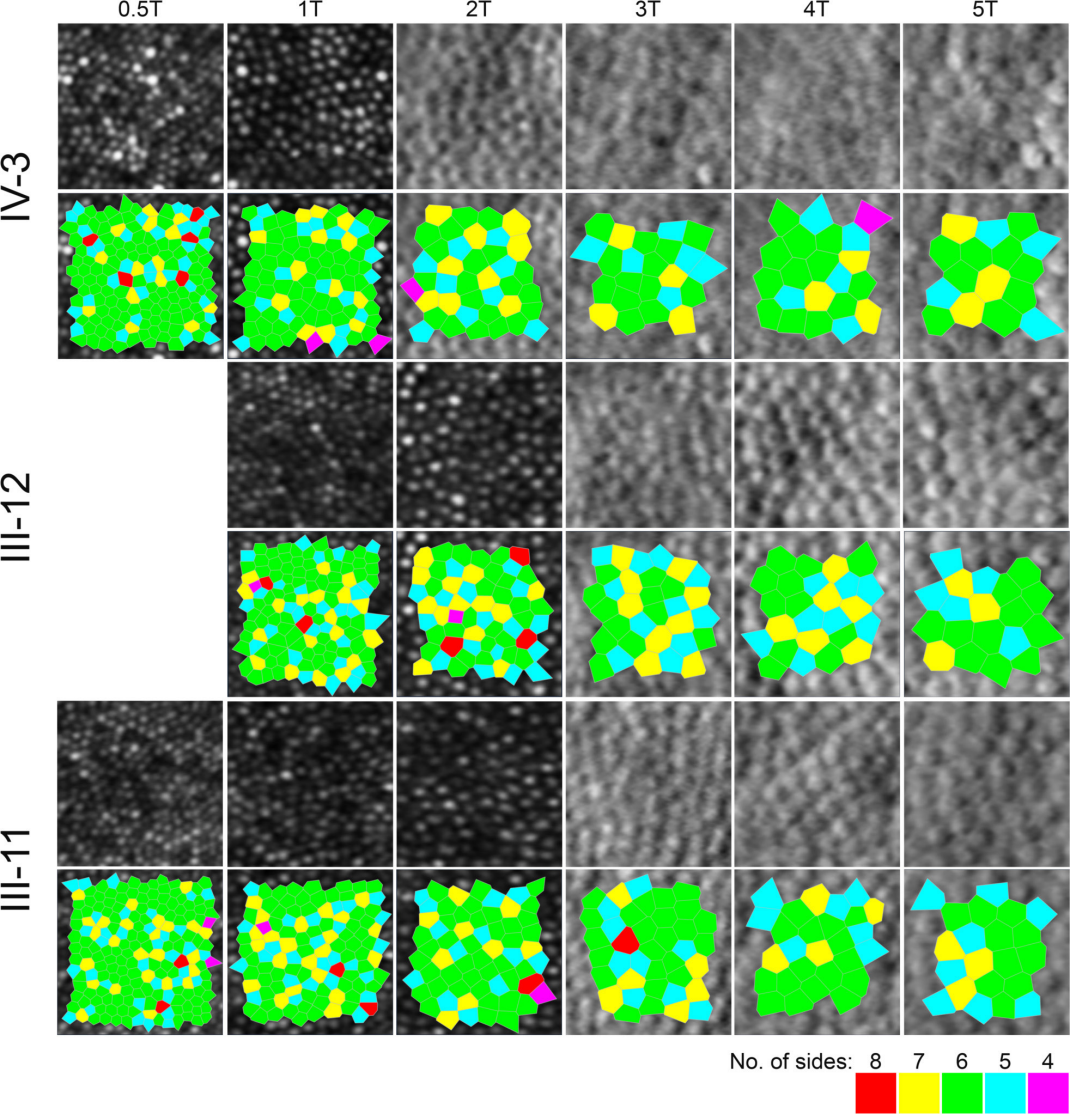
Confocal (outer segments) and split-detection (inner segments) adaptive optics scanning light ophthalmoscope images in the index patient (top row) and her parents (middle and bottom rows) across their temporal meridian. Each crop is 55 microns square. Below each row, the bound cells that were used to derive the cone densities as shown in online supplemental file 7, are illustrated by the Voronoi tessellation.

Confocal images were used for establishing cone densities in densely packed eccentricities closer to the fovea, while split-detection images were used beyond two degrees eccentricity for the affected child (index patient, IV-3) and three degrees for her adult parents. Peak cone density (PCD) for IV-3 (14 years old at the time of the visit) was 109 600 cones/mm^2^. Both parents’ foveal cones were not fully resolved, therefore their foveal centres were identified by means of overlaying their AO montages on other imaging modalities with the aid of blood vessel landmarks and the foveal pit. These locations served as the starting point for identifying ROIs across the temporal meridian for all three family members where possible (figure 4). All cone density values are summarised in online supplemental file 7.

Cooper and colleagues reported normative cone density values using an identical AOSLO system in a cohort of 20 unaffected individuals, including 6 children and 14 adults (Cooper *et al*, temporal meridian, eccentricity bins as per online supplemental file 7).^27^ Due to the different eccentricities reported in Cooper *et al* compared with our analysis for 3° and 5° temporally, we opted to report the next available eccentricity bin (ie, less dense) in order to make our comparisons conservative (ie, 3T in our case is approximately 900 µm and 5T is approximately 1500 µm). Despite the conservative comparison, the cone densities of the index patient were reduced by approximately 20% to as much as 50% compared with the normative density values. The cone densities of the minimally symptomatic mother (III-12) were also outside the previously reported normative cone density ranges. Conversely, the cone densities of the unaffected father were within the normative range.

#### ERG

ERG was performed in three members of the family (IV-3, III-2 and III-12). Full-field recordings identified a rod-cone pattern of dysfunction in symptomatic individuals (IV-3 and III-2), with evidence of peripheral macular dysfunction using large-field PERG. Electrophysiology testing, including large-field PERG was normal in the index patient’s minimally symptomatic mother (III-12) (online supplemental files 8 and 9 for ERG recordings of IV-3 and III-2, respectively).

10.1136/bjophthalmol-2020-318034.supp8 Supplementary data

10.1136/bjophthalmol-2020-318034.supp9 Supplementary data

## Discussion

We describe the clinical and genetic findings in 11 affected individuals, from a four-generation British family with adRP. Genetic analysis, including WGS, identified a deletion of 1-bp in exon 6 of the *RDH12* gene (c.763delG). This variant is predicted to introduce a frameshift leading to a termination codon, 23 codons downstream of the variant. All family members who reported nyctalopia demonstrated classical features of a rod-cone dystrophy.

Quantification of foveal retinal structure by means of SD-OCT was undertaken in two children and three adults (online supplemental file 1). The index patient (IV-3) when compared with their age-matched OCT of unaffected brother had a mildly thinner retina overall. This thinning was further attributed to the ONLT, which ranged from 3.4% to 4.0% thinner (right and left eyes, respectively). This appears to be in keeping with the relatively mild form of the autosomal dominant mode of inheritance compared with the severe, autosomal recessive disease-causing variants in this gene.^15^

Similarly the age-similar adult relatives also demonstrated a comparable thinner retinal thickness. This was attributed to a thinner ONL of up to 20% compared with the unaffected father of the index patient.

Unfortunately, direct comparisons of our data to normative values in the literature is difficult as the majority of studies using Spectralis SD-OCT imaging in adults and in children report average values for the 1 mm diameter central foveal subfield of the Early Treatment Diabetic Retinopathy Study template. The reason we opted to measure a single point foveolar location was to be confident that we had largely excluded the Henle’s fibre layer and obtain a true measure of just the ONL thickness.^31^ This would enable more accurate OCT measurements in the context of our en face cone photoreceptor quantification by means of AOSLO imaging because the ONLT measure would be accounted for by cone (rather than both cone and rod) photoreceptor nuclei.

To the best of our knowledge, we identified only one study with a cohort of 83 unaffected children aged 5–15, that reported the 5th and 95th percentiles of foveal TRT; these were 196.7 µm and 250.2 µm, respectively.^32^ Therefore, the index patient in our study appears to have a foveal TRT within the normal range. That normative study did not measure foveal ONLT. A recent study (although using a different OCT system) reported the foveal ONL thickness in 42 unaffected adults to be ranging from 128.1 μm to 97.7 μm for their right eyes and from 126 μm to 98.2 μm for their left eyes.^33^ Therefore, in our study affected adult family members appear to be on the lower end of the ONLT normative spectrum, being borderline normal in this measure, consistent with the milder form of the genotype. That normative study did not measure foveal TRT. There was another study by a group from Singapore, with the ethnicity of the population studied being different to our family and also a different OCT system employed; nevertheless their normative foveal TRT ranged from 272.2 μm to 230.5 μm.^34^ In individual III-2 who was significantly affected, their foveal TRT in both eyes were well below the lower limit for normal. Interestingly, the minimally symptomatic and affected index patient’s mother III-12 was at the lower end of the normative range.

As our study was retrospective there were a number of limitations, including the lack of dense OCT volume scans. Consequently our data had a wide inter-scan distance (236 microns), which on occasions may have led to an underestimation of the foveal TRT and ONLT when the fovea was missed. The patients also had variable lengths of follow-up.

The index patient’s mother’s (III-12) had normal funduscopy despite being minimally symptomatic at the age of 40, when compared with all other affected family members. Interestingly though, subtle, subclinical endo-phenotypes were detected using short-wavelength FAF and AOSLO (figure 2 and online supplemental file 7). FAF imaging, revealed a hyper-autofluorescent area temporal to the fovea which was stable over the 1.5 years of follow-up. Her cone density measurements across all temporal meridians were consistently lower for any given eccentricity, when compared with her unaffected, age-similar husband, and importantly to a published normative data set imaged using an identical AOSLO system.^27^ It is of note that OCT imaging failed to reveal an abnormality in foveal ONL thickness, neither did ERG. These findings are more in keeping with highly variable expressivity rather than non-penetrance of the disease. The disease-causing variant therefore co-segregates with a varied level of phenotypic expression in this family.

All affected individuals had peripapillary sparing of RPE, usually seen in Stargardt disease,^35 36^ autosomal recessive bestrophinopathy^37 38^ and first reported by Garg and colleagues in *RDH12* associated LCA in 2017, and more recently by others.^39 40^

Panel-based screening for genes known to be associated with retinal dystrophy was performed on the initial three affected family members who presented (including the index patient), while in nine individuals the specific familial variant was screened for by bidirectional Sanger sequencing. One affected family member (III-2) was also subject to WGS, enabling us to minimise the possibility that non-coding or structural variants undetectable by targeted gene panel testing were causative.^13 14^ In light of this evidence we suggest that the *RDH12*:c.763delG is a pathogenic allele, and results in rod-cone dystrophy, but with variable expression.

The variant detected here is located within the penultimate exon of *RDH12*, and the resulting termination codon (TGA) is located 16bp upstream of the final splice donor site (online supplemental file 10). According to accepted models of non-sense mediated decay (NMD),^41 42^ we therefore propose that this variant will lead to an aberrant transcript that may survive NMD and lead to a protein product with a mutant 23 amino acid residue C-terminal sequence, replacing 62 residues of the wild-type protein. Similarly, the adRP-associated *RDH12* variant reported by Fingert and colleagues^18^ (c.778delG) leads to the same reading-frame, although occurring five codons downstream. This is also the case for the variant recently identified by Sarkar and colleagues c.759delC; p.(Phe254Leufs*24).^19^ Other (recessive) pathogenic variants in exon six have been described, including missense, non-sense, splice-site and insertions/deletions.^43^ *RDH12*:c.806_810del p.Ala269Glyfs*2, is one such allele, which has been reported in multiple families sharing the same haplotype.^11^ Although this variant occurs downstream of the adRP-associated variant reported here, and so would also be expected to evade NMD, it leads only to the incorporation of a single glycine residue before premature termination, with the loss of the C-terminal 48 residue peptide. We therefore hypothesise that simply truncating the protein within the classical NMD-escaping region of the gene will lead to loss of functional protein, with heterozygous carriers of such a variant being unaffected, but the reading-frame specific C-terminal peptide (SRRHGRGRRPACTAPWLRAWSP) introduced as a result of the c.763delG (online supplemental file 10), in addition to similar peptides produced by c.759delC or c.778delG, all act as dominant alleles, with a toxic effect on the rod photoreceptors. A similar toxic allele effect has been suggested previously in dominant *RGR-*chorioretinopathy with a similar C-terminal frameshift mutation,^44 45^ and we believe that this represents the most likely mechanism of disease, although this remains to be molecularly evaluated.

10.1136/bjophthalmol-2020-318034.supp10 Supplementary data

A therapeutic approach for this dominant allele would include antisense oligonucleotide (AON) therapy. Carriers of recessive alleles are unaffected by RDH12 disease. Hence being able to switch off the dominant gain-of-function mutant allele using AON targeting would leave affected patients expressing 50% of normal RDH12 like an unaffected parent of an RDH12 LCA child.

Interpreting the consequences of genetic variants can be difficult when variants in a gene exhibit both dominant and recessive disease, within the guidance set out by the American College of Medical Genetics.^46^ It is important to correctly apply the interpretation guidelines with the predicted dominant *RDH12-*disease association, since we note it is gain of function and not loss of function. Co-segregation of the variant and disease state is one key characteristic that helps to provide confidence in causality, however diagnosing the presence of a disease phenotype may not always be straightforward. Here, this detailed family study, including the index patient, her parents, grandparents and extended family have enabled us to conclude that *RDH12*:c.763delG is pathogenic, despite the mother, who carries this variant, being minimally symptomatic, and has a normal fundus appearance using conventional techniques. This study also highlights the benefits of detailed clinical phenotyping (using FAF and AOSLO), along with extended family studies, especially when attempting to interpret the significance of genetic variants that may cause inherited retinal disease, a group of conditions known to show incomplete penetrance and variable expression. Asymptomatic patients who are genetically at risk who may not manifest any remarkable abnormalities could be referred to a specialist centre with expertise in advanced AO retinal imaging and analytics.

This study presents data to support the pathogenicity of a novel *RDH12* variant, c.763delG; (p.Val255Serfs*23), as a cause of autosomal dominant rod-cone dystrophy with variable expression, and presents detailed clinical characteristics.

## Data Availability

All data relevant to the study are included in the article or uploaded as supplementary information.
