# Supplementary material for: Novel disease-causing variant in RDH12 presenting with autosomal dominant retinitis pigmentosa
Source: Br J Ophthalmol. 2021 May 24;106(9):1274–81. doi: 10.1136/bjophthalmol-2020-318034 (PMC9411907; doi:10.1136/bjophthalmol-2020-318034)
Supplement: Supplementary data [file bjophthalmol-2020-318034supp007.pdf]

Supplemental File 7. Cone densities along the temporal meridian in proband (IV-3), minimally symptomatic mother (III-12) and unaffected father (III-11) compared to the relevant reported normative values from Cooper et al. 2016.[27]

Eccentricity from foveal centre	0.5T	1T	2T	3T	4T	5T
Eccentricity bin from Cooper et al.	150 µm	300 µm	600 µm	1000 µm	1200 µm	1600 µm
IV-3	71.6 ± 5.8	41.8 ± 1.0	20.7 ± 1.1	14.5 ± 0.5	11.4 ± 1.6	10.7 ± 0.7
III-12	na	52.6 ± 2.2	28.8 ± 2.3	18.3 ± 0.7	14.8 ± 0.7	11.5 ± 0.8
III-11	78.5 ± 0.7	53.8 ± 1.1	32.2 ± 1.0	21.8 ± 1.1	16.6 ± 0.3	13.8 ± 0.5
Cooper et al.	90.7 ± 13.0	61.8 ± 8.4	39.0 ± 5.7	24.6 ± 5.0	18.6 ± 2.2	14.4 ± 1.7

Units are $\times 10^3$ cones/mm^2 ± standard deviation.

T – degrees temporal; na – not available.
