# Supplementary material for: Novel disease-causing variant in RDH12 presenting with autosomal dominant retinitis pigmentosa
Source: Br J Ophthalmol. 2021 May 24;106(9):1274–81. doi: 10.1136/bjophthalmol-2020-318034 (PMC9411907; doi:10.1136/bjophthalmol-2020-318034)
Supplement: Supplementary data [file bjophthalmol-2020-318034supp001.pdf]

Supplemental File 1. Foveal Total Retinal Thickness and Outer Nuclear Layer Thickness for five family members.

Individual Family member	Age (years)	Foveal TRT (μm)		Foveal ONLT (μm)	
		OD	OS	OD	OS
IV-3 (affected)	15	241	240	114	121
IV-4 (unaffected)	15	254	255	118	126
% diff for IV-3		-5.3%	-6.1%	-3.4%	-4.0%
III-2 (affected)	40	219	215	98	99
III-12 (affected)	40	232	240	97	102
III-11 (unaffected)	44	251	254	114	121
% diff for III-2		-13.6%	-16.6%	-15.1%	-20.0%
% diff for III-12		-7.9%	-5.7%	-16.1%	-17.0%

The percentage difference (% diff) was calculated as 100 × (difference/mean). The affected individuals always have thinner foveal thickness (indicated by the minus sign) than their age-matched unaffected family members.

TRT –Total Retinal Thickness; ONLT – Outer Nuclear Layer Thickness; OD – oculus dextra; OS – oculus sinistra.
