# Supplementary material for: Novel disease-causing variant in RDH12 presenting with autosomal dominant retinitis pigmentosa
Source: Br J Ophthalmol. 2021 May 24;106(9):1274–81. doi: 10.1136/bjophthalmol-2020-318034 (PMC9411907; doi:10.1136/bjophthalmol-2020-318034)
Supplement: Supplementary data [file bjophthalmol-2020-318034supp003.pdf]

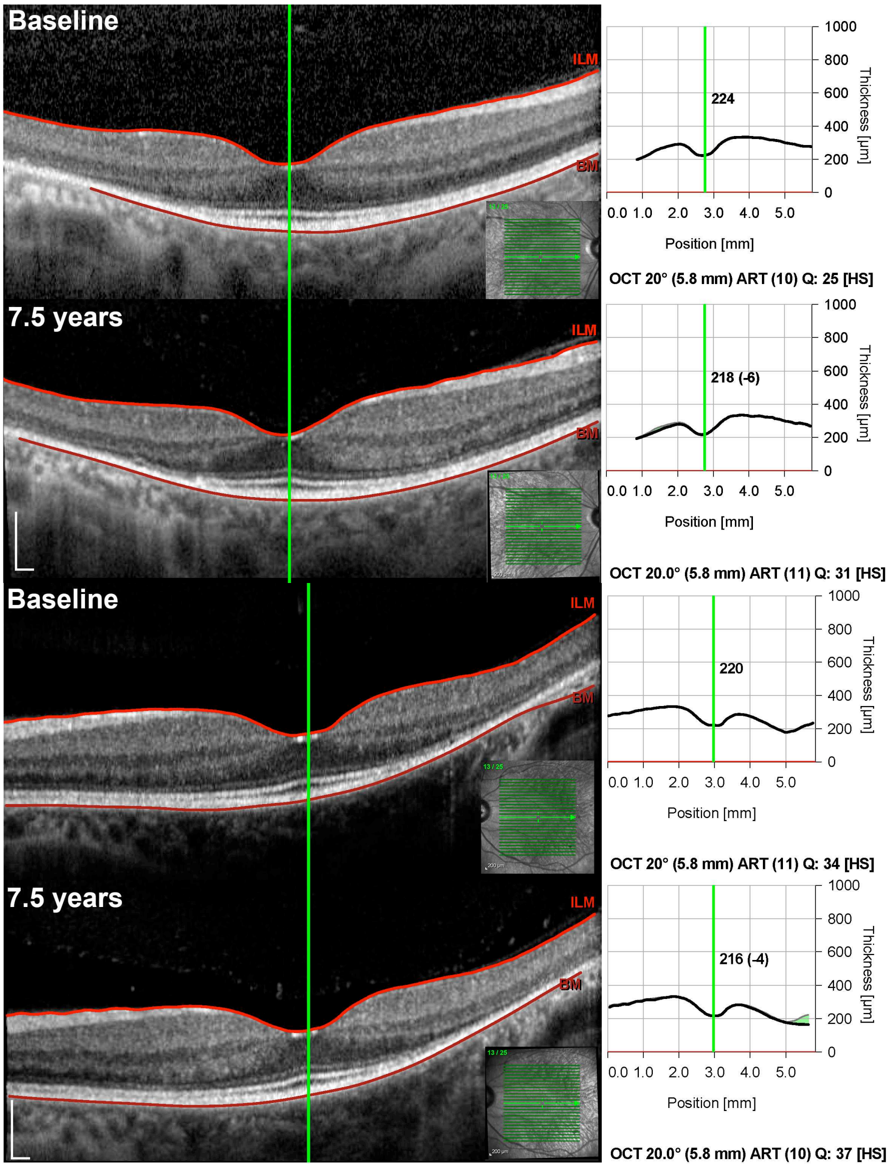

Supplemental File 3. Affected family member III-2 (36 years old at baseline). Longitudinal analysis (7.5 years) of foveal Total Retinal Thickness (TRT) in both eyes. OCT scans obtained in follow-up mode and registered to the baseline visit. Small retinal thickness loss for right (6µm) and left eye (4µm). ILM; Internal Limiting Membrane, BM; Bruch's Membrane. Scale bars, 200 µm.
