# Supplementary material for: Novel disease-causing variant in RDH12 presenting with autosomal dominant retinitis pigmentosa
Source: Br J Ophthalmol. 2021 May 24;106(9):1274–81. doi: 10.1136/bjophthalmol-2020-318034 (PMC9411907; doi:10.1136/bjophthalmol-2020-318034)
Supplement: Supplementary data [file bjophthalmol-2020-318034supp004.pdf]

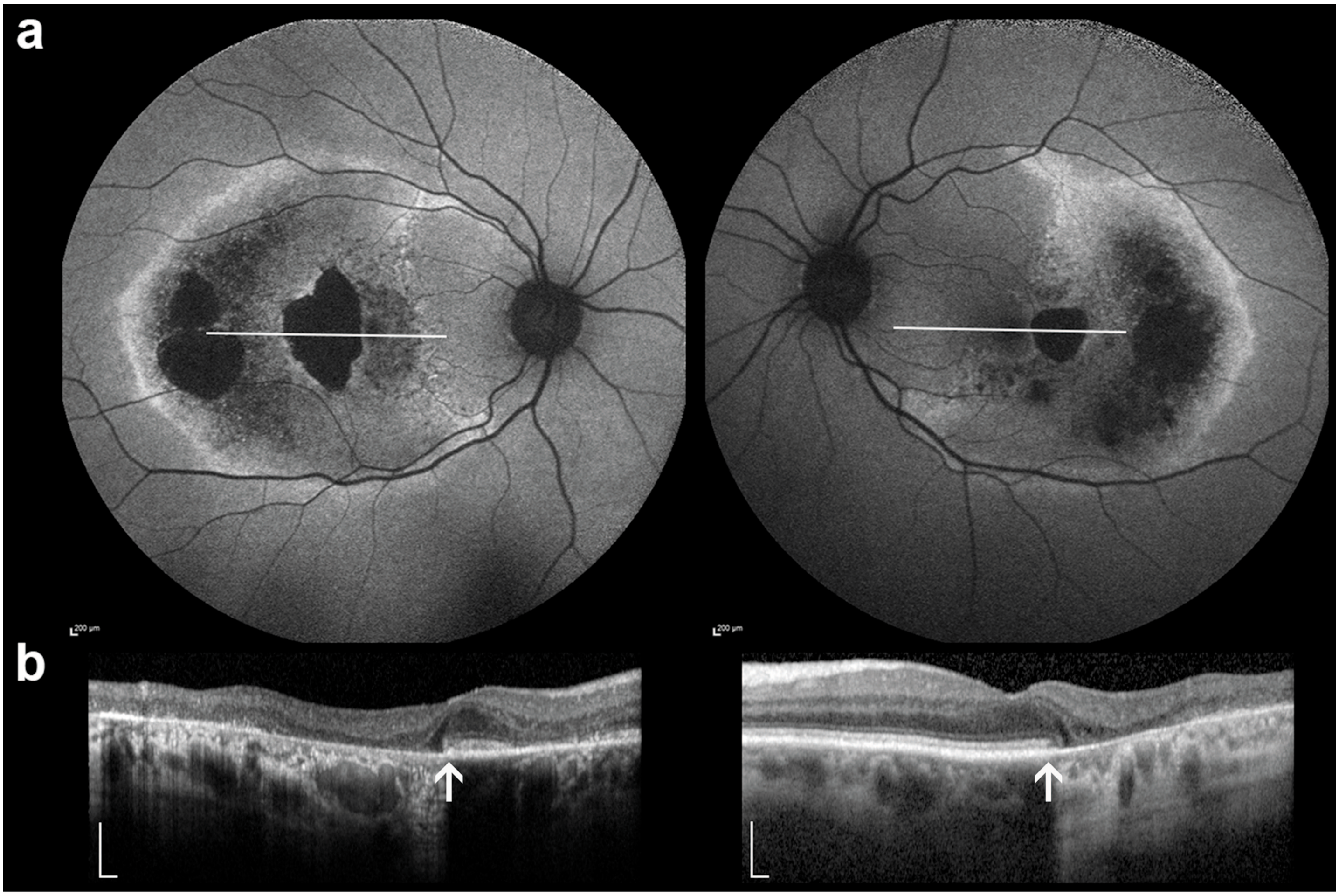


Supplemental File 4. Fundus autofluorescence of individual II-7 a) shows extensive parafoveal and temporal macula hypo-autofluorescence bilaterally b) The corresponding OCT scans taken at the white lines in 4a, show the point at which the outer retina has been lost temporal to the fovea (arrows).
