# Supplementary material for: Novel disease-causing variant in RDH12 presenting with autosomal dominant retinitis pigmentosa
Source: Br J Ophthalmol. 2021 May 24;106(9):1274–81. doi: 10.1136/bjophthalmol-2020-318034 (PMC9411907; doi:10.1136/bjophthalmol-2020-318034)
Supplement: Supplementary data [file bjophthalmol-2020-318034supp005.pdf]

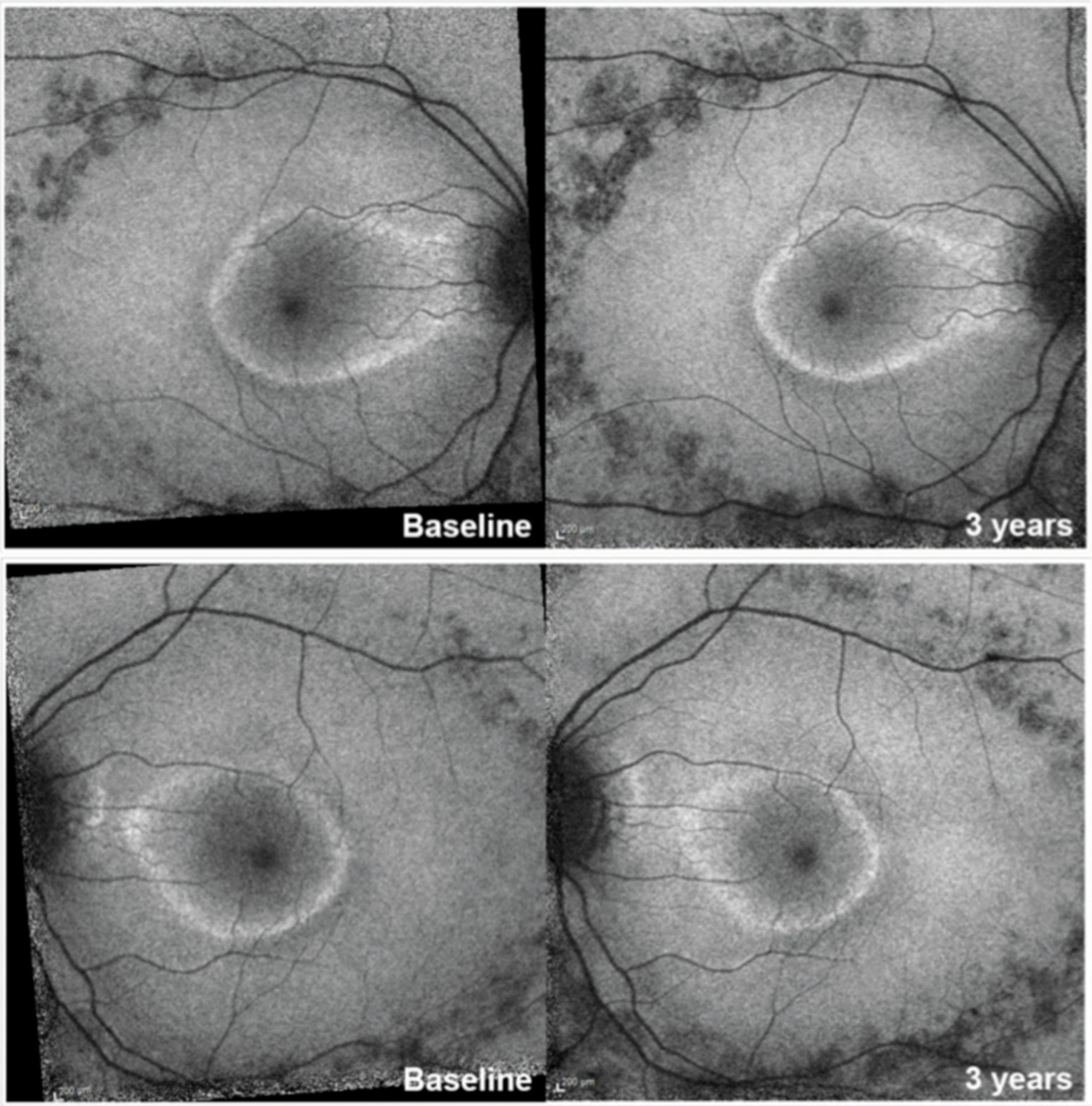

Supplemental File 5. Fundus autofluorescence (FAF) of proband IV-3 (12 years old at baseline). Longitudinal progression (3 years) of short-wavelength FAF 35 degrees wide for the right (top row) and left eye (bottom row). Baseline images have been spatially registered to the follow-up visit. A gradual constriction of the parafoveal hyper-autofluorescent rings and an increase in the area of hypo-autofluorescence along the temporal arcades and at the temporal macula in both eyes at 3 years compared to baseline.
