# Supplementary material for: Novel disease-causing variant in RDH12 presenting with autosomal dominant retinitis pigmentosa
Source: Br J Ophthalmol. 2021 May 24;106(9):1274–81. doi: 10.1136/bjophthalmol-2020-318034 (PMC9411907; doi:10.1136/bjophthalmol-2020-318034)
Supplement: Supplementary data [file bjophthalmol-2020-318034supp006.pdf]

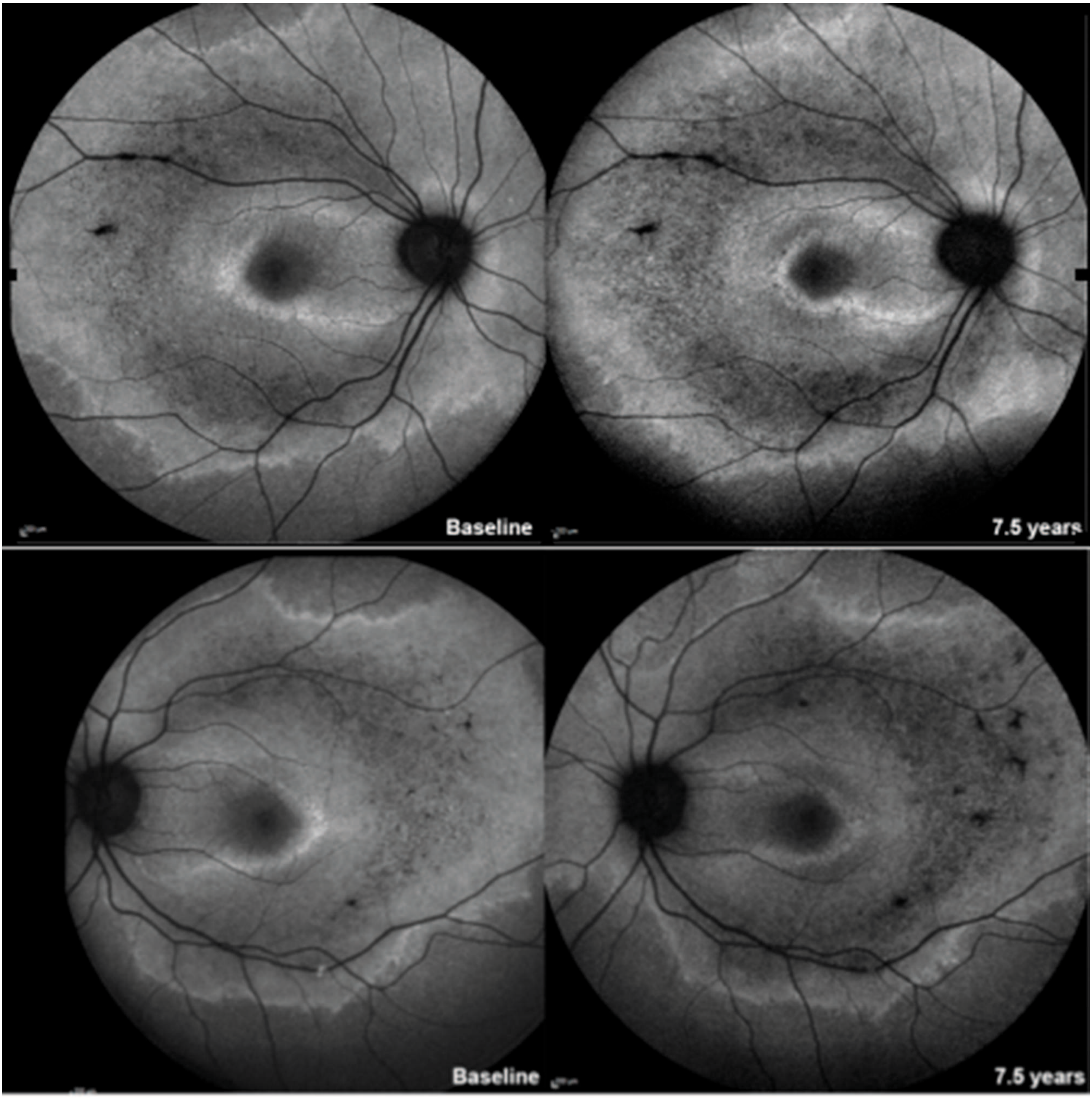

Supplemental File 6. Fundus autofluorescence (FAF) of affected family member III-2 (36 years old at baseline). Longitudinal progression (7.5 years) of short-wavelength FAF, 55 degrees wide, for the right (top row) and left eye (bottom row). Baseline images have been spatially registered to the follow-up visit. Gradual constriction of the parafoveal hyper-autofluorescent rings and an increase in the area of perifoveal hypo-autofluorescence at the temporal macula in both eyes at 7.5 years compared to baseline.
