# Supplementary material for: Novel disease-causing variant in RDH12 presenting with autosomal dominant retinitis pigmentosa
Source: Br J Ophthalmol. 2021 May 24;106(9):1274–81. doi: 10.1136/bjophthalmol-2020-318034 (PMC9411907; doi:10.1136/bjophthalmol-2020-318034)
Supplement: Supplementary data [file bjophthalmol-2020-318034supp008.pdf]

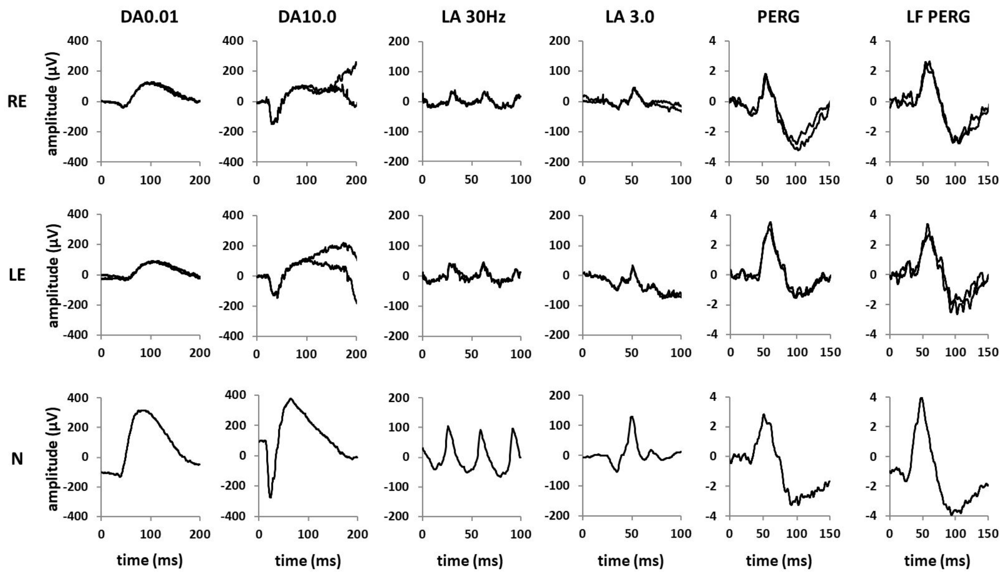

Supplemental File 8. Full-field ERGs and standard field and large field PERGs from the right (RE) and left (LE) eye of the proband (IV-3) are compared with recordings from a representative unaffected control subject (N). Full-field ERGs are consistent with a rod-cone dystrophy. The standard PERG P50 is normal in keeping with preserved macular function. Large field PERG P50 is of similar amplitude to the standard response, consistent with retinal dysfunction over paracentral (eccentric) areas.
