# Supplementary material for: Novel disease-causing variant in RDH12 presenting with autosomal dominant retinitis pigmentosa
Source: Br J Ophthalmol. 2021 May 24;106(9):1274–81. doi: 10.1136/bjophthalmol-2020-318034 (PMC9411907; doi:10.1136/bjophthalmol-2020-318034)
Supplement: Supplementary data [file bjophthalmol-2020-318034supp009.pdf]

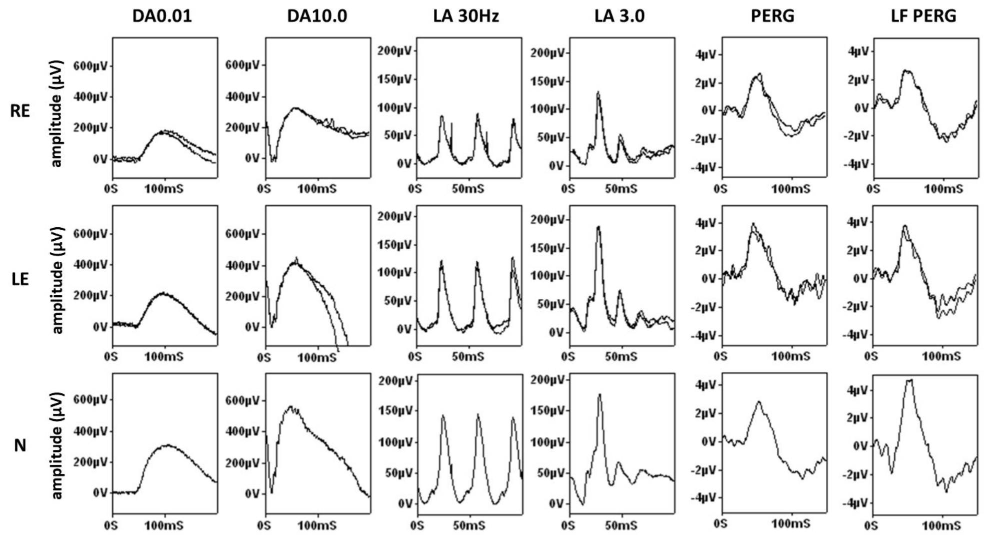

Supplemental File 9. Full-field ERGs and standard field and large field PERGs from the right (RE) and left (LE) eye of subject III-2 compared with recordings from a representative unaffected control subject (N). Full-field ERGs are slightly smaller in the right eye than the left eye remain with the normal range bilaterally. The standard PERG P50 is normal but the lack of enlargement to a doubled stimulus field suggests dysfunction over paracentral (eccentric) macular areas bilaterally.
