# Supplementary material for: Novel disease-causing variant in RDH12 presenting with autosomal dominant retinitis pigmentosa
Source: Br J Ophthalmol. 2021 May 24;106(9):1274–81. doi: 10.1136/bjophthalmol-2020-318034 (PMC9411907; doi:10.1136/bjophthalmol-2020-318034)
Supplement: Supplementary data [file bjophthalmol-2020-318034supp010.pdf]

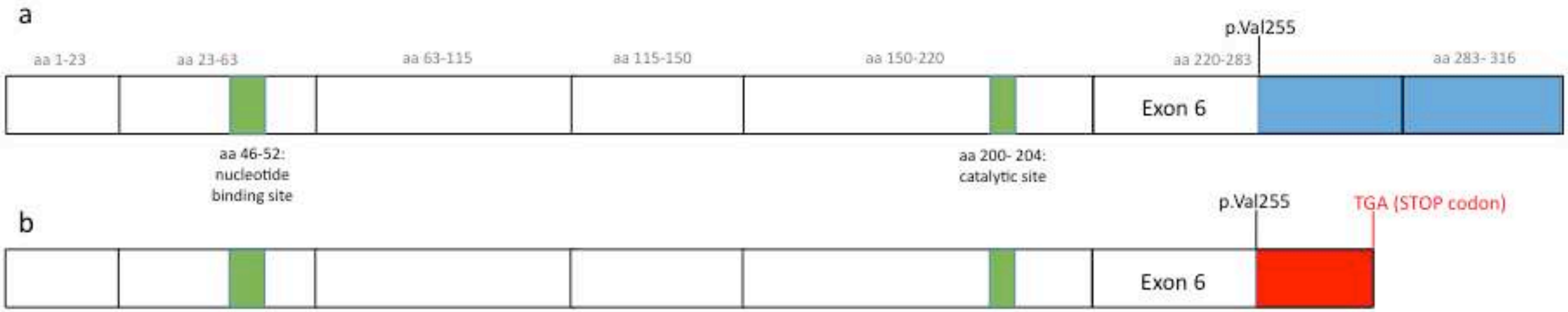

Protein sequence:
Wildtype
MLVTLGLLTSFFSFLYMVAPSIRKFFAGGVCRTNVQLPGKVVVITGANTGIGKETARELASRGARVYIACRDVLKGESAASEIRVDTKNSQVLVRKLDLSDTKSIRAFAEGFLAEEKQLHILINNAGVMMCPYSKTADGFETHLGVNHLGHFLLTYL
LLERLKVSAPARVVNVSSVAHHIGKIPFHDLQSEKRYSRGFAYCHSKLANVLFTRELAKRLQGTGVTTYAVHPGVVRSELVRHSSLLCLLWRLFSPFVKTAREGAQTSLHCALAEGLEPLSGKYFSDCKRTWVSPRARNNKTAERLWNVSCELLGI
RWE (316 amino acids)

Mutant
MLVTLGLLTSFFSFLYMVAPSIRKFFAGGVCRTNVQLPGKVVVITGANTGIGKETARELASRGARVYIACRDVLKGESAASEIRVDTKNSQVLVRKLDLSDTKSIRAFAEGFLAEEKQLHILINNAGVMMCPYSKTADGFETHLGVNHLGHFLLTYL
LLERLKVSAPARVVNVSSVAHHIGKIPFHDLQSEKRYSRGFAYCHSKLANVLFTRELAKRLQGTGVTTYAVHPGVVRSELVRHSSLLCLLWRLFSPFSRRHGRGRRPACTAPWLRAWSP (276 amino acids)

Supplemental File 10. Amino acid sequences of the wild-type and mutant proteins showing the sequence of the c-terminals (wild-type, blue; mutant, red; aa, amino acid).
a) Schematic of the wild-type full length RDH12 protein including the c-terminal amino acid sequence (blue). The unfilled boxes represent the seven coding exons with green boxes representing domains highly conserved in short chain dehydrogenases/reductases, of which RDHs are a subfamily.
These domains are the NADP(H) cofactor binding site at amino acids 46-52 (GANTGIG) and catalytic site at amino acids 200-204 (RDVLK).[8]
b) Schematic of the mutant RDH12 protein predicted to evade NMD resulting from the c.763delG variant in exon 6. The frameshift results in incorporation of a non-native c-terminus (22 amino acid residues, red) and a premature stop codon leading to a truncated protein.
